# KIASORT: Knowledge-Integrated Automated Spike Sorting for Geometry-Free Neuron Tracking

**DOI:** 10.1523/JNEUROSCI.1594-25.2026

**Published:** 2026-06-05

**Authors:** Kianoush Banaie Boroujeni, Thilo Womelsdorf, Sabine Kastner

**Affiliations:** ^1^Princeton Neuroscience Institute, Princeton University, Princeton, New Jersey 08544; ^2^Department of Psychology, Vanderbilt University, Nashville, Tennessee 37212; ^3^Department of Psychology, Princeton University, Princeton, New Jersey 08544

**Keywords:** flexible probes, large-scale neural data analysis, Neuropixels, nonhuman primate neurophysiology, spike sorting

## Abstract

Modern high-density neural recordings demand spike-sorting algorithms that can handle diverse probe geometries and complex, neuron-specific drift, yet existing methods often rely on rigid geometric assumptions and one-dimensional drift models. Here, we introduce KIASORT (Knowledge-Integrated Automated Spike Sorting), a geometry-free approach for per-neuron drift tracking. KIASORT builds channel-specific sorting models from a hybrid linear–nonlinear sample-sorting stage, using representative template banks or supervised classifiers. These channel-specific models then sort spikes by independently tracking each neuron, unconstrained by probe layout. Biophysical simulations showed that even submicron probe displacements induce neuron-specific waveform distortions that standard drift models cannot correct. In ground-truth benchmarks with heterogeneous, neuron-specific drift, KIASORT outperformed Kilosort4 in recovering high-quality units while maintaining real-time performance on standard CPUs. Its robustness was further illustrated on both primate and mouse data. KIASORT combines automated sorting with manual curation in a unified graphical interface, offering a complete and user-friendly spike-sorting platform. The software is freely available at https://kiasort.com.

## Significance Statement

Accurate spike sorting remains a fundamental challenge in systems neuroscience, particularly as recording technologies advance toward simultaneous monitoring of thousands of neurons and the next generations of recording probes. Current methods often rely on rigid assumptions about probe geometry and uniform drift patterns for different neurons which often fail in real-world recordings. We introduce Knowledge-Integrated Automated Spike Sorting with capability to track neurons in a geometry-free framework, as a fundamentally new approach that addresses these critical limitations with many use cases which are not supported by other existing methods.

## Introduction

Brain neural communication relies on the spiking activity of single neurons. The accurate estimation and identification of the neuronal sources generating each spike from extracellular recordings remain a challenge. Such source identification is an essential step toward understanding neural circuit dynamics and compartmental computations in the brain (Einevoll et al., 2012; Hagen et al., 2015). With advances in electrophysiological recording technologies, particularly high-density silicon probes such as Neuropixels that enable simultaneous recordings of large neuronal populations, the need for robust and automated spike-sorting methods has grown significantly (Jun et al., 2017; Steinmetz et al., 2021).

Traditionally, spike sorting involved three main steps: detection, feature extraction, and clustering (Quiroga et al., 2004). Early methods mostly relied on manual clustering of low-dimensional features of spike waveforms, such as principal component analysis (PCA), and further manual adjustments (Harris et al., 2000; Quiroga et al., 2004), facing limitations in scalability, variability, and subjective bias (Einevoll et al., 2012; Pillow et al., 2013; Chung et al., 2017). To resolve these challenges, more automated spike-sorting algorithms have been introduced (Pachitariu et al., 2016; Rossant et al., 2016; Chung et al., 2017; Jun et al., 2017; Yger et al., 2018). These algorithms often use a template-matching paradigm optimized for high-density laminar probes. Some examples include Kilosort, SpyKING Circus, MountainSort, and IronClust. Notably, Kilosort, in particular Kilosort4, has been shown to outperform earlier algorithms through graph-based clustering and drift correction using piecewise rigid probe registration (Pachitariu et al., 2024).

Key challenges remain, however. Despite these advances, algorithmic cores still rest on rigid biophysical and statistical assumptions that real recordings frequently violate. First, spikes are modeled as linear, low-rank superpositions of fixed templates embedded in Gaussian noise; yet extracellular waveforms depend nonlinearly on neuronal morphology, electrode orientation, and tissue conductivity, producing polarity inversions, amplitude-only drifts, and clipped artifacts beyond any low-rank linear model (Gold et al., 2006; Buzsáki et al., 2012). Second, *k*-nearest-neighbor graphs constructed in Euclidean PCA space remain warped by residual common-mode fluctuations and amplitude shifts during drift. These anisotropic distortions, when judged by linear distance metrics, can still fracture sparse units into filaments or cause dense units to bleed together (Hilgen et al., 2017; Yger et al., 2018). Finally, most methods correct only piecewise rigid motion along the *z*-axis; oblique insertions, wide Utah arrays, or angled Neuropixels 2 shanks introduce shear and rotation that remain unmodeled (Pachitariu et al., 2024). In addition, recent motion-correction benchmarks have also shown that estimating and interpolating drift from extracellular traces remains a limiting step, even for modern Kilosort-style pipelines, particularly when motion is nonrigid, locally heterogeneous, or poorly described by a single depth displacement (Garcia et al., 2024; Watters et al., 2025; Windolf et al., 2025).

Here, we introduce Knowledge-Integrated Automated Spike Sorting (KIASORT), a novel algorithm designed to overcome these limitations. KIASORT comprises three main modules: (1) representative multichannel waveform sample extraction, (2) per-channel sorting of those samples using a hybrid approach combining linear and nonlinear dimensionality reduction, and (3) full-recording spike assignment. We first show through biophysical modeling that even subtle drift patterns not aligned with the probe shank produce significant waveform changes that conventional methods leave uncorrected. KIASORT therefore handles each neuron independently through a geometry-free, neuron-based tracking system that builds channel-specific template banks or classifiers from sampled clusters. This per-neuron approach identifies neurons across multiple channels and supports the cross-channel transfer of spikes to a reference channel for drift correction without prior assumptions on the drift type allowing channel-specific models to adapt to nonlinear waveform changes.

We evaluated KIASORT using simulated ground-truth multichannel datasets mimicking real-world recording conditions, including realistic noise profiles, neuron-specific drift, nonlinear waveform transitions, and waveform heterogeneity. The simulation results show that KIASORT outperformed Kilosort4, across several metrics achieving 5–10% higher sorting precision and identified 5–15% more high-quality single units (F1 score > 0.75) under pronounced neuron-specific drift and nonlinear waveform changes. On the geometry-based benchmarks used for Kilosort4, KIASORT showed slightly lower performance but still ranked highest after Kilosort4 among existing methods.

## Materials and Methods

### Denoising

To remove any shared noise across a large number of recording channels, we applied a percentile-template regression that preserves neural spikes waveforms intact. The denoising can be GPU-accelerated when supported. For each raw signal $X\in \;R^{N\times M},$ with *N* channels and *M* time samples, we first compute the percentile at each time point as follows:

$$g=\mathrm{percentile}(X,p)\;\in \;R^{1\times M}\;,\;(1)$$

where *p* is the percentile value used to form the noise template. By default, it is set to 50 (meaning 50% of channels need to show the noise pattern) but can be adjusted by user if the noise pattern is different in their datasets (e.g., when noise is present on less than half of channels). Then each template is moved to the center as follows:

$$\tilde{g}=g-\bar{g}\;\bar{g}=\;\frac{1}{M}\overset{M}{\underset{\;j=1}{\sum }}\;g_{j},\;(2)$$

and each channel data centered as follows:

$$X_{c}=X-\bar{X},\;\bar{X}=\;\frac{1}{M}\overset{M}{\underset{\;j=1}{\sum }}\;X_{j}\in \;R^{1\times M},\;(3)$$

then the correlation between each channel and the noise template as follows:

$$r_{n}=\;\frac{X_{c,n\;}{\tilde{g}}^{T}}{\|X_{c,n}{\|}_{2}\;{\left\|g\right\|}_{2}},\;n=1,\cdots ,N\;(4)$$

for each channel *n*, the algorithm finds a regression gain as follows:

$$\beta_{n}=\;\frac{X_{n}\;g^{T}}{\;{\left\|{\tilde{g}}_{2}\right\|}^{2}},\;(5)$$

and an amplitude gain as follows:

$$\alpha_{n}=\;\frac{\sigma_{g}}{\;\sigma_{x_{n}}},\;(6)$$

where $\sigma_{g}$ is the standard deviation of the noise template and $\;\sigma_{x_{n}}$ is the standard deviation of channel *n*. For all channels exceeding both an amplitude gain threshold and a correlation threshold, the algorithm subtracts the noise from the signal as follows:

$$\;\mathrm{if}\;\alpha_{n}>a_{th}\;\&\;r_{n}>\rho_{th}\;:\;X_{n_{d}}=X_{n}-\beta_{n}g,\;(7)$$

where *ρ_th_* is the correlation threshold, by default set to 0.4, and *α*_th_ is the amplitude gain threshold indicating a fraction of root-mean-square amplitude, by default set to 0.05 to conservatively ignore normal spiking activity (Franke et al., 2010; Jun et al., 2017).

#### Signal quality check

To assess channel quality, we compared the power in the spike band (1.5 kHz < f < 4.5 kHz) with the power in the low-frequency band (0.5 kHz < f < 1.5 kHz) and the high-frequency band (4.5 kHz < *f* < *f_s_*/2). We then normalized the power in each band by its bandwidth and computed a ratio that indicates whether a channel is dominated by low-frequency or high-frequency noise.

$$R_{w}=\;\frac{\max(W_{\mathrm{low}-\mathrm{band}},\;W_{\mathrm{high}-\mathrm{band}})}{W_{\mathrm{middle}-\mathrm{band}}}.\;(8)$$

The ratio is then transformed to scale to the bands as follows:

$$\mathrm{rati}o_{w}=\;\;R_{w}\exp\;\left(\frac{R_{w}-1}{2}\right).\;(9)$$

If the ratio is too high, it indicates that the signal is dominated by either low- or high-frequency noise without prominent midband power (Hill et al., 2011; Banaie Boroujeni et al., 2020). We set the ratio threshold to the smaller of a fixed value (3 by default) and the mean ratio across all channels plus three standard deviations. We evaluated this approach on simulated multichannel datasets as described above and on real recordings from nonhuman primates (Fig. S1). For the spike sorting, any channel not passing this quality check will be excluded from spike detection and waveform extraction.

#### Sample extraction

For the sample-extraction phase, the algorithm selects stratified *t*-second chunks of signal (default *t* = 1 s) across the recording for all channels, *X*(ti:ti + t). In the current implementation, *M* = 300 chunks are extracted by default from regularly spaced temporal intervals, with a random start point within each interval, and users can reduce or increase *M* depending on the recording duration, expected firing rates, and available compute (the more sample extracted during this phase, the better outcome of sorting will be). It is worth noting that very low-firing units below ∼0.1 Hz may be missed. Once all sample chunks have been extracted, they are concatenated into signal Xsample, which is then used for subsequent sample spike detection.

#### Spike detection

For each recording channel, we detect all spikes that exceed a threshold set by multiplying a scale factor by the median absolute deviation (MAD) of that channel's signal. The algorithm first bandpass filters the data using a configurable Butterworth bandpass filter (500–6,000 Hz by default in the current configuration, with user-adjustable values for other sampling rates or recording conditions) and then calculates the MAD threshold as follows:

MAD=median|x(t)–median(x(t))|.(10)

The initial threshold is set by default to 5.5 × MAD in the current configuration. During sample extraction, KIASORT performs an initial detection pass, excludes detected peaks from the noise estimate, and then uses a slightly conservative adjusted threshold (0.9 of the initial value) to collect representative sample spikes. During full data sorting, the stored sample-phase threshold defines the main detection threshold, while additional reserve peaks at half this value are retained for possible cross-channel lookup. Users can adjust the scale factor in the GUI or by scripting depending on their data. Spikes exceeding the relevant threshold are detected. For detected spikes that occur too closely in time, only the one with the highest peak amplitude is retained. The minimum interval between spikes is set to 0.425 ms by default, but if the distribution of interspike intervals (ISIs) shows its largest peak between 0.425 and 0.75 ms, the interval is adaptively increased to match that peak and prevent double counting of multiphasic spikes with widely but regularly spaced peaks.

#### Waveform extraction

For each detected spike on a given channel, KIASORT extracts a full multichannel waveform window around the most extreme positive or negative peak (default full window, 1 ms). The local spatial neighborhood is configurable. In the default code, waveforms are extracted from the center channel plus seven channels on either side (15 channels total), but this can be adjusted in the GUI or configuration file; when probe-coordinate metadata are available, the same concept can be expressed as a local spatial radius rather than a fixed above/below channel count. In practice, users should choose a neighborhood that covers the unit's local footprint, commonly ∼100–200 µm for dense linear probes, while wider spacing or nonlaminar geometries may require a different value. During the sample-extraction phase, all extracted waveforms are saved for every channel. During the main sorting phase, only waveforms for accepted reference-channel assignments are saved, and waveform saving is optional.

#### Handling overlapping spikes

When two spikes occur very close in time (i.e., when the difference between consecutive main spike indices is less than L), the extracted windows overlap. During the sample-extraction phase, we consider only spikes that have no overlapping spikes detected within their waveform window on any neighboring channels. This prevents splitting a single neuron into multiple clusters due to consistent overlaps with other neurons during clustering.

For the main data-sorting step, however, to compensate for overlapping spikes, the algorithm first computes the overlap length based on the ISI and then applies a smoothly varying sigmoid window to attenuate the contribution of the overlapping regions from neighboring spikes. If the time difference between consecutive spikes is Δ*t* (in samples) and Δ*t* < *L*, the overlap length is computed as follows:

$$L_{\mathrm{overlap}}=0.5(L-\Delta t).\;(11)$$

For each possible overlap length *i* (with 1 ≤ *i* ≤ *L*), we find a sigmoid window as follows:

$$\sigma (i)=\;\frac{1}{1+e^{\left(x-i\right)}},\;(12)$$

where *x* is sample point form spike center. Two modified windows are then defined: the left-side window equals *σ(i)* over its left half and 1 over its right half, while the right-side window equals *σ(i)* over its right half and 1 over its left half. Depending on which side the overlap occurs, the corresponding left- or right-side window is multiplied elementwise with the waveform.

### Sorting samples

In this step, the algorithm loads the sample data previously extracted and saved for each channel. Each channel is then processed individually, spike waveforms are preprocessed, and features are extracted. Spikes are clustered, and the accepted clusters are used to build representative templates and, when selected, to train a classifier. After processing each channel, the algorithm unifies clusters across channels and assigns a reference channel to each cluster.

#### Waveform preprocessing and feature extraction

For each channel, spike waveforms were extracted as *T* time samples centered on detected threshold crossings across C channels. For each detected spike *i*, the extracted waveform is a matrix *W_i_* ∈ *R^C^* *^×^* *^T^* spanning *±τ* ms around the spike peak, where *τ* is half of the clustering spike duration. To prepare for dimensionality reduction, each waveform matrix is first flattened into a one-dimensional vector *vec(W_i_)* ∈ *R^1×C.T^* and multiplied by a Hamming window function to weight stronger on the central portion of the spike:

$$w_{i}=\mathrm{vec}(W_{i})⊙h,\;(13)$$

where h ∈ R*^1×^*^C·T^ is the Hamming window and ⊙ denotes elementwise multiplication.

We used a hybrid approach combining linear and nonlinear dimensionality reduction to form a feature space that captures both global and local structures in the spike waveforms. First, the algorithm applies PCA to the preprocessed waveforms:

$$X_{PCA}=(W-\mu_{W})V,\;(14)$$

where W ∈ *R^N^* ^×^ ^(*C*·*T*)^ is the matrix of *N* (number of spikes) flattened spike waveforms, *µ* is the mean waveform vector, and V ∈ *R*^(*C*·*T*)*×n*^ is the first *n* PCs.

For the nonlinear part, the algorithm uses Uniform Manifold Approximation and Projection (UMAP; McInnes et al., 2020) as follows:

$$X_{\mathrm{UMAP}}=\mathrm{UMAP}(W⊙H,n_{\mathrm{comp}}),\;(15)$$

where *H*∈ *R^1×C.T^* is the Hamming window applied to all waveforms. *H* is formed by concatenating C individual Hamming windows, each of length T sample points, and *n*_comp_ is the number of UMAP components. While PCA largely represents the global variance across the entire waveform, UMAP is more weighted by the central portion of each spike to contain spatial information. It preserves both local and global topological structures and the relative distances between waveforms by projecting them into a low-dimensional nonlinear manifold. In the implementation, the Hamming taper is applied only to the waveform vector used for UMAP. PCA, template generation, and SVM/MLP features are computed from the untapered flattened waveform. Thus, the taper reduces the influence of low-SNR waveform edges on UMAP's local neighborhood graph without removing amplitude information used for final matching or classification. The waveform duration is configurable, and its default selection (1 ms) and Hamming taper were validated on the benchmark data to yield the highest performance.

The final feature space used for clustering is constructed by concatenating the normalized UMAP components, the first three PCA components, and the peak amplitude values, as follows:

$$X_{\mathrm{clustering}}=\mathrm{norm}([X_{\mathrm{UMAP}},X_{\mathrm{PCA}},A]),\;(16)$$

where each feature dimension is min–max normalized to the range [0*,*1].

#### Density-based clustering

The normalized feature matrix *X*clustering ∈ R*^N^* ^×^ *^D^* is then used for clustering using an iterative DBSCAN method (Ester et al., 1996). For each sample *x_i_* ∈ R*^D^*, DBSCAN defines a neighborhood as follows:

$$N(x_{i},\varepsilon )=\{x_{i}:\;\|x_{j}-x_{i}\|\leq \varepsilon \},\;(17)$$

and a point is considered a core point if

$$\left|N(x_{i},\varepsilon )|\geq \mathrm{MinPts},\;(18\right)$$

where MinPts is the minimum-points parameter in DBSCAN, set according to a minimum firing rate of 0.1 spikes/s, and bounded between 0.1% (lower bound) and 1% (upper bound) of the total data points.

For clusters with ISI violation ratios exceeding a threshold (indicating potential mixing of multiple units), we recursively apply DBSCAN with adjusted parameters until either the clusters satisfy the refractory-period constraint or a maximum recursion depth of four is reached. After all recursions, we unify the labels, and any noisy clusters or points identified as noise are assigned a “–1” label. The refractory/ISI criterion is therefore used only to decide whether a cluster should be split again. It is not used to label final units as good by construction; final ISI violation, contamination, amplitude, waveform, and cross-correlogram measures remain available for curation and quality assessment.

#### Parameter estimation for DBSCAN

To optimize DBSCAN parameters, we use a knee detection method (Satopaa et al., 2011) to estimate optimal epsilon. We use *k* as the number of datapoints, and then compute the *k*-distance for each point *x_i_* as follows:

$$D_{k}(x_{i})=d_{1}(x_{i},NN_{k}(x_{i})),\;(19)$$

where *NN_k_(**x**_i_)* is the *k*th nearest neighbor using Manhattan distance. After sorting distances, we detect the “knee” in the sorted distance curve, $\;D_{\left(1\right)}\leq \;D_{\left(2\right)}\leq \ldots \leq D_{\left(n\right)}$, by maximizing the perpendicular distance to the line connecting the first and last points as follows:

$$\delta_{i}=\;\frac{\left|\Delta_{y}(i-1)-\;\Delta_{x}\;(D_{\left(i\right)}-\;D_{\left(1\right)})\right|}{\left\|\Delta \right\|},\;(20)$$

where Δ*_x_* is *n* − 1, Δ*_y_* is *D*_(*n*)_ − *D*_(1)_, and Δ is (*n* − 1, *D*_(*n*)_ − *D*_(1)_). The epsilon is then set as follows:

$$\;\varepsilon =D_{(i*)\;}:\;i^{*}=\mathrm{argma}x_{1\leq i\leq n}\delta_{i}.\;(21)$$

#### Cluster merge

When the clusters are labeled for each channel, the algorithm postprocesses these clusters to avoid overspill. For clusters with a mean waveform *w_s_* ∈ *R^M^* *^×^* *^T^* and spike density of time series ds, initial labels *l_0_*, and sample count ns, the algorithm performs the following steps:

First, for each pair of clusters (*i,j*), it computes the cross-correlation between their mean waveforms and identifies the maximum correlation coefficient:

$$\left(c_{ij},\tau_{ij})=\mathrm{argmax}\;\tau x\mathrm{corr}(w_{i},w_{j};\tau ),\;(22\right)$$

where *c_ij_* is the maximum correlation coefficient peak and *τ_ij_* is the time lag at which the peak value occurred.

It computes the amplitude variability as follows:

$$a_{ij}=\;\mathrm{mean}\left(\frac{\left|A_{i}-\;A_{j}\right|}{\max(A_{i},\;A_{j})}\right),\;(23)$$

where *Ai* is the maximum absolute amplitude of *w_i_.* Then it computes the correlation of spike density as follows:

$$r_{ij}=\mathrm{corr}(d_{i},d_{j}).\;(24)$$

After computing these metrics, clusters can be merged using two strategies: stringent merge and loose merge. In the stringent merge, clusters are merged if they show low amplitude variation and high zero-lag cross-correlation between their mean waveforms:

$$a_{ij}<0.05\;\max(A_{i},\;A_{j}),\;\;c_{ij}>0.9,\;\tau_{ij}=0.$$

For the loose merge, clusters are merged if they satisfy the following:

$$a_{ij}<0.15\;\max(A_{i},\;A_{j}),\;\;c_{ij}>0.85,\;\tau_{ij}\neq 0,$$

where the nonzero lag criterion accounts for temporal misalignment of multiphasic spike waveforms.

Lastly, the algorithm checks for drift-based merge where clusters show as follows:

$$a_{ij}<0.15\;\max(A_{i},\;A_{j}),\;r_{ij}<-0.8,\;c_{ij}>0.85,$$

to merge split clusters caused by pronounced spatial drift.

For all merge operations, the resulting cluster's ISI violation rate (i.e., the percentage of ISIs within the refractory period) must not increase by >0.1% (Hill et al., 2011; Kadir et al., 2014).

#### Unifying clusters across channels

After clustering spikes on all channels, we assign a reference channel to each cluster. The reference channel is the channel on which the cluster's mean waveform shows its highest absolute peak and determines which channel-level template bank or classifier will be used during the sorting stage. For each cluster on a given channel, the reference channel can be either the original channel (in which case the cluster is retained) or a different channel (in which case it is transferred). When a cluster's reference channel differs from its original channel, the algorithm checks all channels detecting that cluster and enforces consistent reference-channel assignments. If the same cluster is assigned reference channels on two or more channels (defined by >50% overlap in spike indices), the channels are unified, and only the one with the highest peak value is retained. Finally, if a cluster's reference channel is not its original channel, the algorithm iteratively confirms that the cluster exists on the reference channel; if not, it relocates the cluster to the ultimately retained reference channel.

#### Template and classifier construction

Following unsupervised clustering, KIASORT stores representative template variants for each accepted cluster and can optionally train a supervised multiclass classifier for each channel. We evaluated three classifier types including support vector machine (SVM), multilayer perceptron (MLP), and convolutional neural network (CNN), to compare their performance (Fig. 2*A*). KIASORT supports all three classifiers, but the current full-sorting default is template matching (TM); SVM is the default when the classifier approach is selected because of its computational efficiency, higher accuracy, and roughly 10-fold faster runtime (Fig. 2*B*).

For training, we use either the raw waveforms (for CNN) or the PCA-reduced feature vectors (for SVM and MLP) as follows:

$$\hat{y}=f_{\theta }(x),\;(25)$$

where *x* is the PCA scores of the flattened waveforms or the raw waveforms and *f*_θ_ denotes the classifier with parameters θ. During classifier approach benchmarking, we trained and tested using 50/50 and 75/25 train/test splits; during full-session sorting, the classifier approach is applied only when selected, while the default approach uses the template bank described below.

SVM: We use a degree-3 polynomial kernel (radial-basis-function kernels are also supported, but the polynomial kernel performed best). To exclude potential outliers, the SVM leaves 10% of the sampled spikes out as noise during training.

MLP: The MLP has an input layer; four hidden layers with 2048, 1 024, 128, and 64 units; and an output layer. It uses mean squared error loss, a dropout rate of 0.1, an initial learning rate of 10^−^⁴, and drops the learning rate by a factor of 0.1 every 10 epochs. It is trained on PCA-reduced waveform features.

CNN: The convolutional network comprises four blocks with 16, 32, 64, and 128 filters. Training used a batch size of 128, a kernel size of 3, pooling size of 2, L2 regularization at 10^−^⁴, and matched the MLP’s dropout and learning-rate schedule. In all three classifiers, all hyperparameters were tuned for optimal accuracy.

TM option: In addition to the classifier approach, KIASORT builds a template bank for every accepted cluster. If a cluster contains at least the configured number of examples, its spikes are partitioned into 15 waveform variants by *k*-means in PCA space, and each variant template is the mean multichannel waveform of that subcluster. If fewer examples are available, the cluster mean waveform is repeated across the template slots. During sorting, a detected waveform is matched to all valid template variants on that channel using a weighted waveform distance that emphasizes the central spike samples. The winning template supplies both the predicted label and the variant index used later for scaled subtraction during residual redetection.

### Sorting data

After extracting samples, clustering the sampled spikes, building template banks or training classifiers on each channel's spikes, and unifying spike labels, the algorithm runs the third module to integrate the acquired knowledge and sort the full dataset (Fig. 3).

To make the computation more efficient, multichannel neural recordings are segmented into temporal chunks of duration Tchunk (1-2 min are optimal). To avoid boundary artifacts, each chunk is extended by a small margin Tmargin (2 s), and any spikes detected within this margin are excluded. Similar to the sample-extraction step, raw data in each chunk are bandpass filtered and, when selected by the user, passed through the optional shared-noise suppression and/or whitening steps. For each channel, spikes are detected exactly as in the sampling phase, and waveforms are extracted for each spike. Overlapping spikes are then attenuated using the sigmoid window method described previously.

For each detected spike, the algorithm first checks whether the maximum absolute peak in the local multichannel waveform occurs on the current detection channel. If so, the spike is evaluated on that channel by TM or by the classifier. If the predicted unit is retained on the same channel, the event is stored immediately. If the predicted unit's reference channel differs from the detection channel, the event is withheld from the current channel and added to a lookup list for the reference channel. The algorithm then searches the temporarily stored detections and the subthreshold reserve pool on the reference channel for a time-matched event within 1.5 times the spike-distance tolerance, extracts its waveform, and re-matches it using the reference channel's templates or classifier. A secondary transfer step handles the rare case in which the reference-channel match points to a third channel. To support this cross-channel lookup without excessive memory use or compute overhead, channels are processed in a sliding window by loading classifiers or template banks for the neighboring channels around the current channel. After first-pass assignment, KIASORT further subtracts the scaled matched waveform from the local signal and redetects events on the residual. The residual pass uses the matched template variant when TM is enabled and uses the cluster mean waveform when the classifier approach is used. The full-sorting stage also stores subthreshold reserve peaks detected at half the main threshold; these reserve events are searched during cross-channel transfer so that a spike detected strongly on one channel can still be recovered on its reference channel even if it falls below threshold there. After all channels have been evaluated for each chunk, duplicate detections are removed by label-aware temporal deduplication, unified labels are mapped, and the results are stored in an HDF5 file containing spike indices, labels, channel numbers, amplitudes, features, template variant indices, and optionally multichannel waveforms.

#### The unified GUI platform

KIASORT provides a unified graphical user interface (GUI) that integrates data visualization, inspection, and parameter settings for sorting on its main tab (Fig. 6). The GUI includes three additional tabs:

Channels: Enables users to inspect raw data, manually include or exclude channels, and adjust the MAD threshold via a slider

Curation: Lets users load sorted results and perform post hoc processing; mark unit isolation types, merge or remove channels, realign waveforms, and evaluate waveform similarity; and explore cross-correlograms, presence ratios, ISI distributions, and feature metrics before saving curated outputs (Fig. 6)

Clusters: Offers a sanity-check view of clustering results for each channel, allowing users to fine-tune parameters in cases of atypical datasets

### Biophysical modeling of probe drift effects on spike waveform

We developed a biophysically plausible model to investigate the effects of laminar probes drift on extracellularly recorded action potentials. For this simulation we used a 50-channel linear probe with 25 µm spacing and a high temporal resolution waveform calculation to isolate how small off-axis probe displacements distort extracellular waveforms after idealized depth correction. This modeling was not used for benchmarking and only served to illustrate how nonvertical drifts affect the waveforms in a neuron-specific manner. We model multicompartmental neurons with proper cable dynamics, realistic membrane biophysics, and spatially extended current sources to capture the complex, nonlinear relationship between probe movement and spike waveforms.

#### Multicompartmental neuron models

In the modeling, we considered four distinct neuron morphologies, representing major cell types in cortical circuits, each with biophysically plausible but simplified geometry and compartmentalization (Fig. 4*A*; Table S1):

Pyramidal Neurons (A, D): Large spherical somata (20–22 µm diameter), apical dendrites extending 180–200 µm at 70–100° angles, basal dendrites 100–120 µm long at random orientations, and axon initial segments 180–200 µm in length

Stellate Neuron (B): Medium soma (18 µm diameter), radially oriented dendrites 100–130 µm, axon 150 µm in length

Interneuron (C): Smaller soma (15 µm diameter), shorter dendrites 70–90 µm long, and a thin 1.5 µm axon 120 µm in length

Each compartment was modeled using standard cable theory (Holt and Koch, 1999), in which the membrane potential along each compartment obeys the cable equation as follows:

$$\lambda^{2}\frac{\partial^{2}V_{m}}{\partial x^{2}}=\tau_{m}\frac{\partial V_{m}}{\partial x^{2}}+V_{m}-R_{m}I_{\mathrm{ionic}}(V_{m},t),\;(26)$$

where *λ* is the space constant, *τ_m_* is the membrane time constant (1.0 ms), *R_m_* is the specific membrane resistance, and *I*_ionic_ denotes the nonlinear ionic currents.

#### Membrane ion channel dynamics

The membrane potential dynamics are modeled by nonlinear activation of sodium and potassium channels by adapting biophysically plausible kinetics from the Hodgkin–Huxley equation (Hodgkin and Huxley, 1952; Traub et al., 1991):

$$I_{\mathrm{ionic}}=I_{Na}+I_{K}+I_{\mathrm{leak}}.\;(27)$$

Sodium channel activation followed a sigmoidal relationship with membrane potential:

$$m=\;\frac{1}{1+\exp\;((V_{\mathrm{half}}-V)/k)},\;(28)$$

where *V*_half_ *=* *−*40 *mV* denotes the half-activation voltage and *k* = 6 indicates the slope factor. Channel saturation was incorporated using a hyperbolic tangent nonlinearity:

m=tanh(1.5.m).(29)

Potassium channel activation showed gating and slower kinetics as follows:

$$n={\left(\frac{1}{1+\exp\;((V_{\mathrm{half}}-V)/k)}\right)}^{4},\;(30)$$

with *V*_half_ *=* *−*55 *mV* and *k* = 8.

The resulting compartment-specific currents were modeled for the soma as follows:

$$I_{\mathrm{Na}}=-\;A_{\mathrm{Na}}\;\exp\left(-{\left(\frac{t-t_{AP}}{\tau_{\mathrm{rise}}}\right)}^{2}\right),\;(31)$$

$$I_{K}=A_{K}\;\exp\left(-{\left(\frac{t-(t_{AP}+\delta )}{\tau_{\mathrm{fall}}}\right)}^{2}\right),\;(32)$$

where *t_AP_* is the action-potential timing, *τ*_rise_ and *τ*_fall_ are the compartment-specific membrane time constants, and δ is the delay in potassium-current activation. We also incorporated cell-type–specific differences in the kinetics of these currents with interneurons showing faster kinetics (shorter $\tau_{\mathrm{rise}}$ values) than pyramidal cells (Martina et al., 1998; Bean, 2007).

#### Field potential calculation

We calculated the extracellular potential generated by each compartment using the line source approximation (Holt and Koch, 1999; Gold et al., 2006), which estimates each neuronal segment as a line current source within a homogeneous volume conductor as follows:

$$V(r)=\;\frac{1}{4\pi \sigma }\int_{0}^{L}\frac{I_{\mathrm{line}}(\ell )}{\left|\vec{r}-\;{\vec{r}}^{\prime }(\ell )\right|}d\ell ,\;(33)$$

where *σ* = 0.3 S/m is the extracellular conductivity, *I*_line_ is the transmembrane current per unit length, *r* is the electrode position, and *r′(ℓ)* denotes positions along the compartment of length *L*.

For computational efficiency, this integral was solved analytically for a line segment carrying constant current. For a segment running from ***r***₁ to ***r***₂ at a perpendicular distance *h* from the electrode, the extracellular potential is as follows:

$$V=\;\frac{1}{4\pi \sigma }\;\frac{1}{h_{\mathrm{eff}}}\log\;\left|\frac{\sqrt{h_{\mathrm{eff}}^{2}+\;{\left(L-s\right)}^{2}}\;+(L-s)}{\sqrt{h_{\mathrm{eff}}^{2}+\;s^{2}}-s}\right|.f(h),\;(34)$$

where $h_{\mathrm{eff}}=\;\sqrt{h^{2}+\;d_{\min}^{2}}$, incorporating a minimum effective distance of *d*_min _= 10 µm to avoid singularities, s is the longitudinal projection of the electrode onto the segment, and

$$f(h)=\frac{1}{1+\sqrt{{\left(h/\lambda_{\mathrm{spread}}\right)}^{3}}},\;(35)$$

a spatial-decay function with *λ*_spread_ = 60 µm.

For very short segments or point-current sources, this simplifies to as follows:

$$V=\;\frac{1}{4\pi \sigma }\;\frac{I_{\mathrm{line}}}{d_{\mathrm{eff}}}\;f(d),\;(36)$$

where *d* is the distance from the source to the electrode.

We incorporated a critical biophysical phenomenon, finite propagation velocity of extracellular fields, into our model. Unlike simplified models assuming an instantaneous transmission for neural signals, we account for spatiotemporal signal delays to estimate how electrode drift, combined with propagation time, can distort postdrift spike waveform reconstruction. In the brain tissue, electrical signals travel at roughly 0.1–10 m/s depending on myelination, frequency content, and other medium-related factors (Bédard et al., 2004; Logothetis et al., 2007).

To simulate finite propagation times from neuron to electrode, we consider channel-dependent delays:

$$V_{ch,i}(t)=V_{\mathrm{source}}(t+\Delta t_{i}),\;(37)$$

where $\Delta t_{i}=|d_{i}-d_{\mathrm{source}}|/v$ is the propagation delay between the channel *i* and the nearest channel to the source. Here, *v* is the propagation velocity (5 m/s), which leads to delays of ∼1 µs per-channel distance for high-frequency components (Logothetis et al., 2007). This ensures that signals reach different recording sites with realistic temporal offsets.

#### Probe drift and waveform drift correction

A probe drift was implemented by relocating the probe on the vertical axis by Δ*y* and on horizontal axis for shear effect by Δ*x* displacement (Fig. 4*A*). To avoid any inaccuracies for the reconstruction, we shifted the probe only by multiples of channel spacing (25 µm). In our simulation, we used Δ*y* *=* 50 µm, two different Δ*x* values of 0.5 µm, and 5 µm which represents an angle of ∼0.5̇ and ∼5,˙ respectively.

For each probe, we then correct the drift by simply shifting its channel up or down to match the location of the probe before drift:

$$V_{\mathrm{corrected},i}=V_{\mathrm{drift},\;\;i-\Delta y_{\mathrm{shift}}}.\;(38)$$

All waveforms for each condition were aligned to their peak-amplitude time (*t* = 0) for further comparisons.

To examine the drift-correction efficacy in the context of spike-sorting, we generated 1,000 waveform variants for each neuron before drift and after the drift-correction random amplitude scaling and adding background noise. We then used PCA to reduce waveforms and visualize the separability of clusters in their feature space before and after correction.

### Simulating multichannel extracellular spike data

We simulated multichannel extracellular data by combining background noise and spike data. The simulation generated 30 kHz multichannel recordings on a 64-channel inline probe (25 µm spacing). This simulation was intentionally designed to control each unit's waveform type, firing rate, reference channel, and drift trajectory independently, because the key question was how sorters behave when nearby neurons do not share the same motion pattern. First, we estimated the background signal using spectral features from real Neuropixels recordings. We modulated the FFT of the signal by a coefficient applied to the frequency components and then incorporating a spatial factor so that nearby channels exhibited greater similarity in their low-frequency variabilities. For each recording channel, LFP noise was modeled as the sum across logarithmically spaced frequency bands. For band b, with frequency range [*f*_1*,b*_*,f*_2*,b*_], we compute as follows:

$$X_{b}(f)=\{\begin{array}{c} -\frac{s_{b}}{\;f+\;\epsilon }e^{-i\emptyset (f)}\;\;f_{1,b}\leq f\leq \;f_{2,b}\\ 0,\;\;\mathrm{otherwise}, \end{array}\;(39)$$

where *S_b_* is the scaling factor, *ɛ* is a small constant to avoid singularity at *f* = 0, and *ϕ*(*f*) is a random phase uniformly distributed over (0*,*2*π*) but constrained by a spatial factor that causes nearby channels to exhibit greater phase alignment in their low-frequency bands. The time-domain signal for band b is then obtained via the inverse Fourier transform as follows:

$$n_{ch,\;b}(t)=\{F^{-1}[X_{b}(f)]\},\;(40)$$

where $n_{ch,\;b}$ is the signal for frequency band *b*, at channel *n*. The raw background signal is then found through sum over all frequency bands:

$$N_{ch\;}(t)=\overset{B}{\underset{b=1}{\sum }}\;n_{ch,\;b}(t).\;(41)$$

#### Spike generation

For each channel, we randomly designate 0–3 neurons to show their peak amplitude. Each neuron is then assigned a firing rate *r* from a lognormal distribution (Buzsáki and Mizuseki, 2014) as follows:

$$r∼\mathrm{LogNormal}\;(\mu ,\sigma )\;\;\mathrm{with}\;r\in [r_{\min},r_{\max}],\;(42)$$

where *r*_min_ *and r*_max_ min and max firing rate set between 0.25 and 40 spike/s, respectively. Spike times follow a Poisson process with exponentially distributed ISIs:

$$p(\Delta t)=\;r\;e^{-r\Delta t}\;\Delta t>0.\;(43)$$

For neurons, a refractory period Δ*t*_ref_ = 2 ms is enforced such that

$$t_{k+1}-t_{k}\geq \Delta t_{\mathrm{ref}},\;\;\forall k.$$

We then modeled spike waveforms as monophasic, biphasic, or triphasic shapes to capture variations in morphology and electrode proximity. Monophasic spikes were modeled as follows:

$$V_{\mathrm{mono}}(t)=(1+\delta )\;A\;\exp\left(-\frac{{\left[t-(1+\delta )\;t_{\mathrm{peak}\;}\right]}^{2}}{2[(1+\delta )\sigma^{2}]}\right),\;(44)$$

biphasic spikes as follows:

$$V_{\mathrm{bi}}(t)=(1+\delta_{1})\;A_{1}\;\exp\left(-\frac{{\left[t-(1+\delta_{1})\;t_{\mathrm{peak},1}\right]}^{2}}{2\;[(1+\delta_{1})\;\sigma_{1}^{2}]}\right)-\;(1+\delta_{2})A_{2}\exp\;\left(-\frac{{\left[t-(1+\delta_{2})\;t_{\mathrm{peak},2}\right]}^{2}}{2\;[(1+\delta_{2})\;\sigma_{2}^{2}]}\right),\;(45)$$

and triphasic spike:

$$V_{\mathrm{tri}}(t)=\;\sum_{\;j=1}^{3}\;(-1{)}^{\;j-1}\;(1+\delta_{j})A_{j}\exp\;\left(-\frac{{\left[t-(1+\delta_{j})\;t_{\mathrm{peak},j\;}\right]}^{2}}{2\;[(1+\delta_{j})\;\sigma_{j}^{2}]}\right),\;(46)$$

where *δ_j_* is small random perturbation introducing variability, *A_j_* is the amplitude for each phase extreme, and *t*_peak,*j*_ is time to peak for each extreme. Each spike waveform is further perturbed by multiplicative noise [scaling by 1 + *η* with *η* ∼ N(0, *σ*_mult_^2^)] and an additive noise *ξ*(*t*) ∼ N(0*, σ*_add_^2^):

$$\tilde{s}(t)=[1\;+\;\eta ]\;s(t)\;+\;\xi (t).\;(47)$$

For each spike waveform, we generated a distance-dependent attenuated waveform on neighboring channels. Then, the waveform from its reference channel (where spike showed it largest amplitude) of a spike to recording channel *ch* is attenuated by a Gaussian function:

$$w(\mathrm{ch})=\;\exp\left(-\frac{{\left(ch-c_{\mathrm{ref}\;}\right)}^{2}}{2\;\sigma_{\mathrm{spatial}}^{2}}\right),\;(48)$$

where *c*_ref_ is the reference channel of the spike event and *σ*_spatial_ controls the spatial spread.

For the nonlinear waveform modeling and incorporating voltage-dependent kinetics and curved spike manifolds, we used each of the canonical waveform above and transformed it as follows:

$$s_{nl}(t)=[1+\beta \tanh\;(\gamma s(t))]s(t+\;\delta \tau (t)),\;(49)$$

where $\delta \tau (t)=\;\sum_{m=1}^{M}\;\alpha_{m}B_{m}(t),$ with cubic B-spline basis *B_m_*, and coefficient $\alpha_{m}∼\;N(0,\sigma_{\tau })$, *β* ∼ *U* (*β*_min_*, β*_max_), and *γ* ∼ *U* (*γ*_min_*, γ*_max_), which ranges by ≲30% curvature and ∼20–40 μs timing jitter. In the nonlinear modeling, we ranged the number of units showing nonlinear waveform variabilities from 10, 25, and 50% of all units (Fig. S4).

### Modeling spatial drifts

To model electrode drift, the reference channel *c*_ref_ varies over time in one of the following two ways: smooth (sinusoidal) drift

$$\Delta c(t)=A_{\mathrm{drift}}\;\sin(2\pi f_{\mathrm{drift}}t\;+\;\phi ),\;(50)$$

and step drift

$$\Delta c(t)\;=\{\begin{array}{c} \begin{array}{c} \;\;\\ 0\;\;\;\;\;\;\;\;\;\;t<t_{\mathrm{step}} \end{array}\\ \Delta c(t)\;\;\;t\;\geq \;t\mathrm{step} \end{array},\;(51)$$

with *c*_ref_ (*t*) = *c* + Δ*c*(*t*). The *f*_drift_ indicates the frequency of the drift, and ϕ is the delay phase which is consistent for all units showing drifts. We also considered nonlinear stochastic drift for additional simulations shown in Figure S4 as follows:

$$d\Delta c_{t}=\;-\;\frac{\Delta c_{t}}{\tau_{d}}\;dt+\;\sigma_{d}\;dW_{t}\;(\mathrm{OU}\;\mathrm{diffusion}),\;(52)$$

where $\tau_{d}\;$ is a decay time constant controlling how quickly deviations relax back toward zero, $\sigma_{d}\;$ is the diffusion coefficient indicating the size of the small, continuous fluctuations, $W_{t}$ models a small Wiener process. The final drift model is implemented through adding the Ornstein–Uhlenbeck diffusion process as a nonlinear stochastic component to the continuous sinusoidal drift.

For the benchmark simulations, each unit was assigned either no drift, smooth sinusoidal drift, step drift, or nonlinear stochastic drift depending on the condition. Drift was applied at the unit level by changing the reference-channel position and the distance-dependent amplitude profile over time, with maximum displacement up to 50 µm. This allowed drifting and nondrifting neurons to coexist in the same local channel neighborhood, a case that is difficult for methods that estimate one motion trajectory per spatial bin and apply it to all local traces:

$$c_{\mathrm{ref}}(t)\;=\;A_{\mathrm{drift}}\;\sin(2\pi f_{\mathrm{drift}}t\;+\;\phi )+\;\Delta c_{t}.\;(53)$$

#### Multichannel signal

After generating both spike trains and background LFP activity for each channel, we generated the multichannel signal containing both. The time-domain voltage on channel *ch* at time *t* is the sum of LFP noise and spike contributions and is then calculated as follows:

$$V_{\mathrm{ch}}(t)=\;N_{\mathrm{ch}}(t)+\;\underset{j}{\sum }\;\underset{i}{\sum }\;w_{i}(\mathrm{ch})\;\tilde{s_{j}}(t-t_{i}),\;(54)$$

where the sum runs over all spike events of all unit *s_j_*, occurring at times *t_i_* (Camuñas-Mesa and Quiroga, 2013; Hagen et al., 2015).

#### Benchmarking metrics

To evaluate spike-sorting performance, we matched the detected clusters to ground-truth classes and computed standard metrics. For *K* detected clusters $\left\{ C_{j}\}\begin{array}{c} K\\ \;j=1 \end{array}\right.$ and *M* ground-truth classes $\left\{ T_{i}\}\begin{array}{c} M\\ i=1 \end{array}\right.$, we assigned each cluster to the class by minimizing the Manhattan distance:

$$i^{*}\;=\;\arg\;\min\;d_{1}(c_{j},t_{i}),1\leq i\leq M\;\;\mathrm{where}\;\;d_{1}(c_{j},t_{i})=\;\overset{D}{\underset{d=1}{\sum }}\;|c_{\;j,d}-\;t_{i,d}|.\;(55)$$

For each true class *T_i_*, we computed precision (*P_i_*), recall (*R_i_*), F1 score (*F*_1*,i*_), and accuracy (*A_i_*):

$$P_{i}=\;\frac{TP_{i}}{TP_{i}+FP_{i}},\;R_{i}=\;\frac{TP_{i}}{TP_{i}+FN_{i}},\;P_{i}=\;2\frac{P_{i}\;R_{i}}{P_{i}\;+\;R_{i}},\;A_{i}=\;\frac{TP_{i}+TN_{i}}{TP_{i}+FP_{i}+TN_{i}+FN_{i}},\;(56)$$

where *TP_i_*, *FP_i_*, *TN_i_*, and *FN_i_* denote true positives, false positives, true negatives, and false negatives for Class i, respectively. Under each simulation condition, these metrics are calculated for all classes and compared between KIASORT and Kilosort4 using the Wilcoxon signed-rank test.

## Results

**Figure 1.**
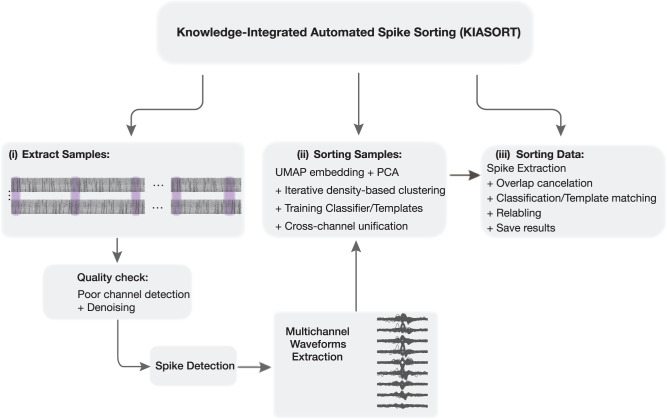
Main diagram of the KIASORT algorithm. The workflow consists of three modules for extracting and integrating knowledge for final sorting of cells. ***i***, Sample extraction. Data are uniformly sampled across the recording to build a representative subset. During this step, channels are screened for poor-quality or silent electrodes, per-channel detection thresholds are defined, and multichannel spike waveforms are extracted for all threshold crossings. ***ii***, Sample sorting. Extracted waveforms are embedded using UMAP and PCA and concatenated into a joint feature space for iterative density-based clustering. Clusters that violate the refractory period or ISI criteria are recursively split, highly correlated clusters are merged, labels are finalized, and either a representative template bank (default) or an optional SVM/MLP/CNN classifier is built from the accepted clusters. Each cluster is then assigned a reference channel based on the site of maximal absolute spike amplitude. ***iii***, Data sorting. The entire dataset is processed in small batches. Spikes are detected and matched to channel-specific templates or classified by the per-channel classifier; spikes whose predicted reference channel differs from the detection channel are transferred and retrieved at their reference-channel. Final outputs including spike times, features, waveforms (optional), channels, and unit labels are stored after processing each batch.

The algorithm consists of three main modules (Fig. 1). The first module (1) screens channel quality, uniformly samples short data chunks across the recording, detects spikes, and extracts a local multichannel waveform around each threshold crossing. The spatial window is configurable; in the current implementation, it is either defined as the detection channel plus *N* channels on each side or select *N* channels that are located within a defined radius. The second module (2) sorts these sampled spike events separately for each channel by clustering waveforms in a hybrid UMAP+ PCA feature space, merging oversplit clusters, assigning a reference channel to each cluster, and building either a bank of channel-specific representative templates (default) or a channel-specific classifier (this option is configurable by users). The reference channel is the channel on which the cluster has its maximum absolute amplitude. The third module (3) sorts the full recording in batches. Each detected spike is first evaluated on the detection channel, retained only if that channel is its local maximum, and otherwise temporarily stored for possible transfer. If the matched unit belongs to another reference channel, the event is looked up on that reference channel and rematched there. This procedure gives each spatiotemporally detected event a single channel-level unit assignment while allowing the same neuron to be tracked across nearby channels.

For the first module, initially, the algorithm performs a brief screening to detect poor-quality or silent channels. Each channel signal (in a short duration of 3 s) is decomposed into multiple frequency sub-bands, and the power in the spike bands is compared with low- and high-frequency bands. We verified this method on simulated data with variable low- and high-frequency noise levels and spike-band activity and show examples from real recordings (Fig. S1). KIASORT also provides optional shared-noise suppression for recordings dominated by common-mode or spatially correlated noise. In the current implementation, this denoising is user-selectable and disabled by default, but it is especially useful in recordings under high noise conditions, so the algorithm can be run with only bandpass filtering and the selected common-reference option when denoising is not required. When enabled, the shared-noise suppression estimates common structure from quiet or low-frequency components and attenuates it while preserving the residual spike waveform used for detection and sorting (see Materials and Methods). In addition, KIASORT also includes a user-selectable preprocessing whitening step to remove shared covariance across channels, implemented similar to that of Kilosort4.

Then a detection threshold is defined for each channel using MAD, and multichannel waveforms are extracted for all threshold-passing events. For each spike that cross the threshold, the sample-extraction stage stores the detection channel, spike time, and local multichannel waveform. Spike events with nearby temporal overlaps on the same or neighboring channels are excluded so that the clustering stage (in the sample-sorting module) learns clean single-spike waveforms. Duplicates across channels are resolved later by the reference-channel and cross-channel unification steps (see Materials and Methods).

### Per-channel clustering and template/classifier construction

Once the spike samples are extracted, the second module (Fig. 1) processes each channel individually, clusters the spikes, and then constructs the channel-level sorting model from the clustered data. In the default approach this model is a bank of representative templates; in the optional classifier approach, it is a supervised multiclass classifier. For each recording channel, the sampled spike waveforms are mapped to their low-dimensional UMAP embeddings and principal components (PCs). In general, PCA captures global linear variance in the waveform data and can effectively suppress local variability (e.g., high-frequency noise) in its first few dimensions. UMAP, in contrast, preserves local nonlinear structure and manifold geometry that linear methods like PCA inherently miss. By concatenating features from both PCA and UMAP, we construct a feature space that integrates global linear trends with the ability to unfold complex, curved manifold structures. This is one of the notable innovations of our approach, which avoids PCA's implicit assumption that clusters are roughly Gaussian (ellipsoidal) in PC space and relaxes those shape constraints by incorporating UMAP. This hybrid feature space improves clustering of arbitrarily shaped or overlapping spike clusters and separating morphologically similar or temporally drifting waveforms (Fig. S2).

**Figure 2.**
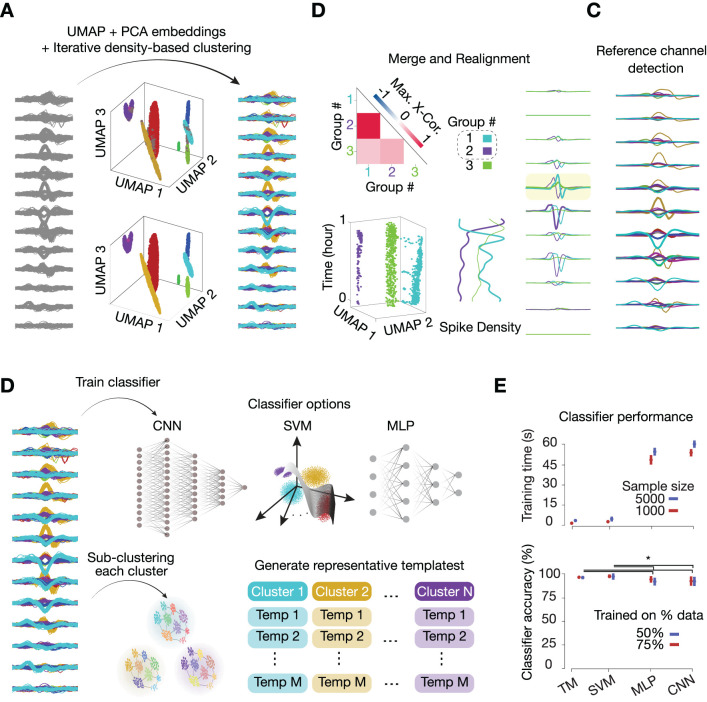
Per-channel spike clustering and template/classifier construction. ***A***, Workflow for each recording channel. Extracted spike waveforms are dimensionality reduced using UMAP and PCA and then concatenated into a single feature space for iterative density-based clustering. Clusters that violate the refractory-period or ISI thresholds are recursively split, and low-confidence spikes (gray) are excluded. ***B***, An example of cluster merging and realignment. Top left, Heatmap of maximum cross-correlations between cluster templates for Groups 1–3 flag Groups 2 and 3 for merging. Bottom left, 3D UMAP embedding over time showing temporal drift and anticorrelated spike-density profiles of Groups 1 and 2. Right, Average waveforms for the three groups. Groups 1 and 2 are merged and assigned to the fifth (yellow shaded) channel. ***C***, After clustering a reference channel is detected for each cluster. Average waveforms are shown for each cluster on neighboring channels. The channel exhibiting the largest absolute peak amplitude is assigned as the reference channel (thick lines). ***E***, Final cluster assignments and waveforms are used to build template variants and, when the classifier approach is selected, to train classifiers (SVM, CNN, or MLP). ***F***, Classifier benchmarking. Top, Training time for TM, SVM, MLP, and CNN on 1,000 (red) versus 5,000 (blue) training sample sizes. Bottom, Classification accuracy for two train/test splits (50 vs 75%). TM outperforms all classifiers and SVM achieve the fastest training and highest accuracy among the tested classifiers. Error bars indicate standard error of the mean, and significant differences (*p* < 0.05) are denoted by asterisks.

After constructing this hybrid low-dimensional feature space of the waveforms, we use an iterative density-based clustering (DBSCAN) approach to detect clusters (Fig. 2*A*). After each DBSCAN round, the spike train of each cluster is tested for refractory-period consistency. Clusters with high ISI violations are treated as likely mixed units and are recursively reclustered with parameters re-estimated from that cluster’s own feature distribution. The ISI violations are used during clustering as a purity trigger, not as a final quality metric. We estimate the DBSCAN radius epsilon by computing each point's *k*th nearest-neighbor distance and selecting the knee of the resulting *k*-distance distribution (see Materials and Methods).

After clustering, to avoid oversplit clusters of spike waveforms (where a single unit with high waveform variability is erroneously divided into multiple clusters), we use a two-tier merging strategy. In the first, stringent merge, we merge clusters showing low amplitude variation (<5% of peak-to-peak between the mean waveforms) and high zero-lag cross-correlation between their waveforms (*r* > 0.9). In the second, looser merge, we merge clusters with looser criteria on their amplitude variation (15%) if their cross-correlation peaks at nonzero lag (to account for temporal misalignment of multipolar spike waveforms) and if clusters show high negative spike-density correlation (*r* < −0.8) to capture splits caused by more pronounced spatial drifts (see Materials and Methods for more details). An example of merge between two misaligned clusters is shown in Figure 2*B*. The two classes show drift (left bottom) and negatively correlated spike density (middle) while showing a high cross-correlation value (top left), all of which decides a merge between the two clusters.

After the merging step, the mean waveform of each cluster is used to define its reference channel. The reference channel is the channel that shows the largest amplitude for that cluster (Fig. 2*C*; reference channels are shown with thicker lines). This reference channel identifies the primary channel to which each cluster belongs, given that multichannel waveforms can be detected across multiple channels. After all channels are processed, the algorithm unifies spike labels across channels, ensuring that each cluster is assigned a single, consistent reference channel across all channels in which it appears. It also assigns cardinal unified labels to all unique clusters detected in the sampled data. This unification step prevents spatial double counting. When the same unit is clustered on multiple nearby channels, KIASORT links the corresponding channel-level clusters based on spike-time overlap and retains the representation with the largest peak amplitude as the reference-channel representation. Other channel-level detections of the same unit are marked for transfer rather than stored as separate units.

After clusters are detected, their labels are used to train per-channel classifiers or to generate representative templates for the full data-sorting module (configurable by the user, with representative templates as the default; Fig. 2*D*). Both approaches use the same clustering and logic in the full data-sorting module. For the classification approach, we considered three classifiers, including SVM, MLP, and CNN, and evaluated their performance against each other. For SVM and MLP, the classifiers were trained on the first 30 PCs of the waveforms, whereas for CNN, the raw waveforms, preserving spatiotemporal structure, were used. In addition to classifier-based sorting, the module supports a TM approach and uses it by default. For each final cluster, KIASORT stores multiple representative waveform variants (15 by default), generated by *k*-means partitioning of spikes within that cluster. Spikes in the full recording are then matched to these variants using a weighted waveform distance. For fair comparison, both classifiers and TM are defined on a training subset of the clustered data and evaluated on a held-out test subset. We trained all models on a subset of the clustered data and tested them on the remaining data, with labels derived from clustering. On average, both TM and SVM significantly outperformed CNN and MLP in accuracy (Wilcoxon test, *p* < 0.001; Fig. 2*E*, bottom). TM showed the fastest overall inference, while among the classifiers, SVM showed ∼10× faster training time (Fig. 2*E*, top). These results were consistent for different sample sizes and test/train splits.

### Per-channel template-/classifier-based spike sorting of the data

**Figure 3.**
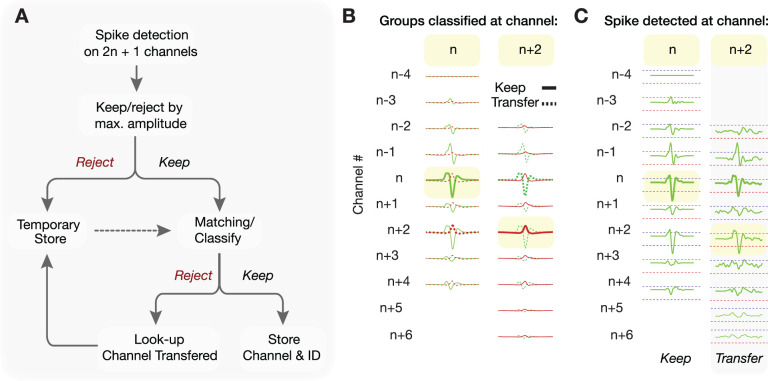
Per-channel template-/classifier-based spike–sorting workflow. ***A***, Flowchart of the two-pass, per-channel sorting procedure. Spikes detected on each channel (2*n* + 1 window) are first retained if their peak amplitude occurs on the detection channel *n* or else sent to a temporary store. Retained spikes enter the channel-specific template matcher or optional classifier: if the predicted unit's reference-channel matches the detection channel, the unit's label is stored; if not, the lookup table directs retrieval of the corresponding stored spike for rematching on its true channel. ***B***, Illustration of class assignments learned on two nearby channels (*n* and *n* + 2). Waveforms whose maximum lies on the local channel are kept (solid traces), while those referencing the other channel are transferred to the channel at which the unit has its maximum (dashed). Here, the green class references channel *n* and the red class references *n* + 2. ***C***, Two example spikes from the same neuron showing maximal amplitude on different channels. The left spike peaks on channel *n* and is correctly labeled in the first pass; the right spike peaks on channel *n* + 2 is initially matched there but is redirected back to channel *n* and in the second pass is correctly transferred to and labeled at channel *n*.

After processing all sample data, clustering spikes, building templates or training per-channel classifiers, and unifying labels, the algorithm executes its third module to sort the full dataset. The full spike sorting is performed in batches. First, each batch is filtered and preprocessed using the same preprocessing options described earlier. Then, the spike threshold for each channel from the sample-extraction phase is applied and spikes are detected on each channel. For each spike detected on a given channel, multichannel waveforms are extracted from the same local neighborhood used during sample sorting. If a waveform exhibits its maximum amplitude on the channel where it was detected, the spike is retained for first-pass matching on that channel; otherwise, it is placed in a temporary store. Retained spikes are assigned by the default TM approach or by the optional channel-specific classifier. After a label is assigned, KIASORT checks whether the matched unit's reference-channel matches the current channel. If it does, the label is accepted and stored. If it does not, the spike is transferred to the lookup/reference channel, the corresponding stored event is retrieved within the temporal tolerance, and the waveform is matched (or classified if selected) again on the reference channel (Fig. 3*A*).

**Figure 4.**
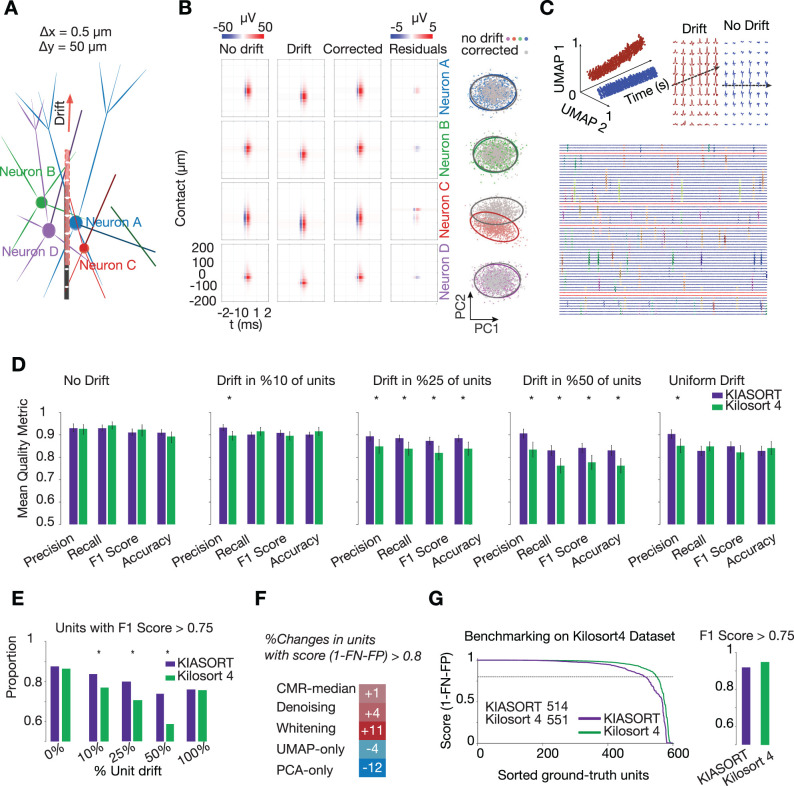
Benchmarking KIASORT performance on simulated datasets with varying drift. ***A***, In silico simulation of laminar recording from multicompartmental neuron models, containing pyramidal cells (neurons ***A*** and ***D***) and interneurons (neurons ***B*** and ***C***). ***B***, Extracellular field for spikes of each neuron are modeled before and after a vertical Δ*y* *=* 50 µm drift and a small horizontal Δ*x* *=* 0.5 µm displacement. Panels from left to right show each neurons’ field before drift, after drift, after drift correction, and the residual from subtracting drift corrected waveform from the waveform before the drift. The rightmost column shows the PCs of waveforms generated for each neuron after drift correction (gray) and before (colored). ***C***, Bottom, Multichannel dataset with spike waveforms with variable shapes (uni-, bi-, and triphasic) associated with morphology of cell types and with different polarities (positive and negative). Sorted spikes are color-coded, and poor-quality channels detected by the algorithm are highlighted in red. An example illustrating two neurons with (brown) and without (blue) temporal drift. Top left, UMAP embeddings show the temporal trajectories of spike features. Top right, Waveform changes over time for the drift versus stable unit. ***D***, Comparison of mean quality metrics (Precision, Recall, F1 Score, Accuracy) between KIASORT (purple) and KiloSort4 (green) at increasing levels of simulated drift. ***E***, Proportion of high-quality (F1 > 0.75) and low-quality (F1 < 0.2) units detected by each algorithm. KIASORT consistently identifies a higher proportion of high-quality clusters across drift conditions. ***F***, Benchmarking on the simulated dataset used by Kilosort4, comparing the number of units with a score (1 − false negative − false positive) > 0.8 (left) and F1 scores (right). In geometry-aware simulations, Kilosort4 yields a slightly higher number of units. ***G***, Effects of preprocessing steps and embedding types on KIASORT performance on Kilosort4 benchmark data (percentage changes).

An example of the same unit representation learned on two nearby channels is shown in Figure 3*B*, showing retained and transferred channel assignments for each class. In this example, the red class has a reference channel at *n* + 2, and the green class has a reference at *n*. This keep/transfer process continuously tracks neurons and enables a per-neuron tracking system: even if drift causes a neuron's maximum peak to appear on a channel different from its original reference channel, the first matching or classification step directs the event to its reference channel for the second round of matching (Fig. 3*C* shows two examples for each condition). For temporal overlaps, the full-sorting stage applies two additional safeguards. First, waveform regions contaminated by nearby spikes are attenuated with a length-adaptive sigmoid window so that the central, most informative spike features dominate matching. Second, after first-pass assignments are made, KIASORT subtracts a scaled version of the matched waveform from the local multichannel signal and redetects spikes on the residual. In the TM approach, subtraction uses the exact matched template variant; in the classifier approach, it uses the cluster mean waveform. This residual pass gives temporally overlapping spikes a second opportunity to be detected and assigned. After all channels are processed for each batch, the results are unified and saved in the HDF5 (Hierarchical Data Format version 5) file format.

### Biophysical simulation, drift correction, and benchmarking

One of the main features that KIASORT is designed based on is its geometry free per-neuron tracking system. Traditionally, drift correction have been done based on geometry of the probe and similar biophysical assumptions about changes in the waveforms of all neurons. Unlike methods using whole-probe drift tracking (Jun et al., 2017), Kilosort4 implements depth-dependent drift-estimation in which the probe depth is divided into overlapping bands, for each of which a drift trace is found, and data alignment is performed on demand through Gaussian kriging interpolation (Pachitariu et al., 2024). While this band-wise approach relaxes the assumptions about whole probe rigid drifts, it still treats motion as a one-dimensional displacement along the probe axis for each band, which often encompasses multiple neurons. Consequently, it does not correct waveform distortions arising from neuronal morphology (e.g., dendritic or axon initial segment current contributions) or from residual horizontal and oblique probe offsets, especially when the heterogeneity between these neural features as well as the proximity of the probe to the recording units increases. To address this, we used two complementary simulation approaches. First, we used a biophysical forward model to illustrate why neuron-specific drift matters in principle, by testing how small nonvertical probe displacements can alter the extracellular waveform of individual neurons differently. Second, we used a separate multichannel benchmark simulation in which neuron-specific drift patterns were implemented explicitly. This second simulation was designed to test the conditions that KIASORT is built for and to provide a direct comparison with Kilosort4 under heterogeneous per-neuron drift conditions.

To better illustrate the limits of such linear-superposition assumptions on drift correction, we built an in silico four-neuron model in which the probe undergoes a 50 µm vertical and a subtle 0.5 µm horizontal displacement (the horizontal shift is 1% of the vertical) and is then perfectly realigned, “corrected,” along the depth-axis (see Materials and Methods; Fig. 4*A*). While tools such as LFPy and MEArec are widely used to generate realistic extracellular recordings, here our goal was more specific: to directly examine how small off-axis probe displacements interact with neuronal morphology to shape the local extracellular field. We therefore used a forward biophysical model to isolate these effects at the level of individual neurons. We modeled the neurons using biophysically realistic multicompartmental models of pyramidal cells and interneurons with different morphologies and kinetics (see Materials and Methods). After drift, channel realignment restores each neuron’s depth position to its baseline by shifting the postdrift waveforms to correct for the vertical displacement (Fig. 4*B*). For each before-drift and after-drift-correction condition, we generated a Gaussian cloud of waveforms to compare their low-dimensional structures. Our results show that by incorporating even as small as <1 µm horizontal displacement, the estimated waveforms can differ substantially, showing notable residuals and distinct low-dimensional structures, particularly for neurons with faster action potential kinetics (Fig. 4*B*; Neuron C). The distortion after drift correction was even worse when the horizontal displacement becomes more notable (>1 µm), which exacerbated residual distortions and led to more distinct low-dimensional structures for all neurons (Fig. S3).

We examined KIASORT performance on both real and simulated datasets. For benchmarking and comparing its performance against the state-of-the-art method, Kilosort4 (Pachitariu et al., 2024), we used simulated multichannel data. Because the main design goal of KIASORT is to handle heterogeneous, neuron-specific drift, we built a complementary benchmark in which nearby neurons could drift differently, remain stable, or undergo nonlinear waveform changes, rather than relying only on existing general-purpose benchmark datasets (e.g., MEArec and Kilosort4; Buccino and Einevoll, 2021; Pachitariu et al., 2024). For our benchmarking, therefore, we implemented per-neuron drift by modulating spike amplitude along the depth axis (see Materials and Methods). First, we generated datasets using biophysically realistic spike waveforms containing both negative and positive spikes with uni-, bi-, and triphasic waveforms representing morphology variabilities and different electrode proximities (see Materials and Methods). We then added background noise by generating a signal whose frequency spectrum matches that of Neuropixels recordings in the mouse cortex, with some channels modeled to mimic the spectral content of silent or poor-quality contacts (Fig. 4*C*). The recording probe for simulation was 64 linearly organized channels and with 25 µm spacing and sampling frequency of 30 kHz. Each neuron could drift up to 50 µm from its original location. Drift was implemented either as continuous Gaussian waveform changes, stepwise neuron displacement, or continuous displacement with nonlinear distortions (see Materials and Methods). An illustrative pair of UMAP trajectories depicts one neuron that drifts and another that remains stationary, highlighting the temporal evolution of waveform space (Fig. 4*C*).

We evaluated both KIASORT (purple) and Kilosort4 (green) on simulated datasets with varying proportions of drifting units, uniformly distributed along the depth axis. For Kilosort4, we optimized the detection threshold and parameters to maximize performance (in general, lowering the threshold tended to introduce false positives). For each detected unit, we computed precision, recall, accuracy, and F1 score (see Materials and Methods). Our simulation results showed KIASORT performing comparably to Kilosort4 on no-drift and uniform-drift conditions while outperforming Kilosort4 in per-neuron drift conditions, with the performance gap widening as drift variability between units became more pronounced (Fig. 4*D*). These performance differences were consistently evident when waveform changes were nonlinear, or drift was implemented nonlinearly (Fig. S4*A*,*B*). Consistent with this finding, the proportion of high-quality units (F1 score > 0.75) detected by each algorithm showed KIASORT identified a significantly higher number of high-quality units, particularly under conditions with greater variability in drift across units (Fig. 4*E*). Overall, while Kilosort4 shows decent performance on conditions with no drift or when neurons within similar localities show consistent drift, our geometry-free and per-neuron tracking shows significantly improved performance, particularly when these assumptions do not hold true in real-world complex neural recordings. In such conditions, KIASORT particularly benefited from UMAP embeddings compared with other preprocessing steps or when using PCA alone (Fig. S4*C*). We also tested the run time for both KIASORT and Kilosort4 on 1 h of 384-channel data. KIASORT showed comparable performance to Kilosort4 when using GPU. On the other hand, while we were unable to run Kilosort4 using only CPU, KIASORT still showed real-time performance with CPU alone (Fig. S4*D*).

We also evaluated KIASORT on the public Kilosort4 benchmark datasets. We first quantified the contribution of different components of our sorter using these ground-truth datasets. Overall, whitening and the use of hybrid dimensionality reduction provided the largest improvements in performance (Fig. 4*F*). On these benchmarks, which are based on smooth waveform libraries and are closer to the assumptions of template-based, geometry-aware sorters, KIASORT remained competitive and overall outperformed all non-Kilosort4 methods included in the benchmark. Kilosort4 retained a modest advantage under its native 1-FN-FP > 0.8 quality score, whereas the methods were more comparable under an F1 > 0.75 criterion that balances precision and recall symmetrically (Fig. 4*G*). Under other geometry-aware drift conditions, KIASORT continued to outperform all other sorters but slightly underperformed Kilosort4 (Fig. S4*E*). This performance gap in geometry-based drift conditions is driven primarily by a modest reduction in recall, while precision remains comparable to Kilosort4. The recall cost shows a weak dependence on cluster size (Fig. S4*F*) and is not explained by spike collisions [i.e., spatiotemporal overlap (Garcia et al., 2022); Fig. S4*G*]. Instead, it arises from KIASORT’s independent channel-wise unification and neuron-based tracking, which is the same design choice that enables geometry-free tracking under heterogeneous per-neuron drift.

### KIASORT performance on mouse and monkey high-density neural recordings

**Figure 5.**
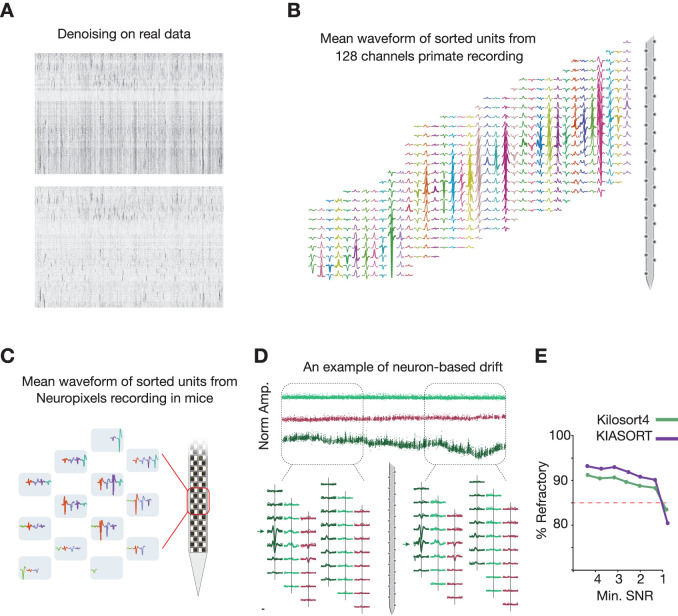
KIASORT results on real data. ***A***, An example of two-step denoising applied to raw primate probe recordings, showing the raw voltage trace with variable artifacts on different channels (top) and the cleaned signal after denoising (bottom). ***B***, Example mean multichannel spike waveforms of units sorted from a 128-channel, dual-shank DBC probe in primate striatum. ***C***, Example mean multichannel spike waveforms of units sorted from a Neuropixels probe recording in the mouse cortex (Allen Institute dataset). ***D***, An example of neuron-based drift in a primate DBC probe: one unit (dark green) exhibits progressive spatial drift over time, while two other units (light green and red) remain stable throughout the recording. ***E***, Fraction of refractory units within different minimum SNR value compared between KIASORT and Kilosort4 for the same session as in ***D***.

We ran KIASORT on datasets recorded from different probes. We used data from the caudate nucleus, anterior cingulate cortex, and lateral prefrontal cortex in the monkey using a dual-shank diagnostic biochip (DBC) probe and datasets from the Allen Brain Observatory (Anon, n.d.), recorded from the mouse visual cortex. In all datasets, we found that our noise removal method could eliminate any shared noise or signal components evident on a high number of channels, even when the noise level was prominent (Fig. 5*A*). Our algorithm yielded ∼2× spike/channel, showing ability to detect even high-firing fast-spiking neurons (fr > 40 spike/s) that often do not show dips in their spike train correlograms. It also was able to track neurons and detect units with different waveform shapes (see Fig. 5*B* for an example). Similar performance was observed for recordings in mouse (Fig. 5*C*). In all datasets, we also documented per-neuron drift as a likely event in neurophysiological recordings. As illustrated in Figure 5*D* as an example, from three highly isolated nearby neurons, only one showed drift with a pattern different from the other neurons. With its geometry-free design and per-neuron tracking sorting system, KIASORT successfully tracked and identified these neurons independently in tested real datasets in recordings from different brain areas, including the striatum, lateral prefrontal cortex, and anterior cingulate cortex which contains neurons with different morphologies (Fig. S5). In the example DBC and Neuropixels recording, KIASORT detected 236 (from 128 channels) and 733 (from 384 channels) and for active channels showed on average 1–2 high-quality isolated single units. In addition, while a direct comparison between KIASORT and Kilosort4 on real data without ground truth is not a valid benchmark, we compared the sorting performance between the two methods. In a recording session from the primate striatum, we computed the spike amplitude on the main channel for both Kilosort4 and KIASORT and defined SNR consistently across methods. We then quantified the fraction of refractory units (defined as those with an ISI violation rate below 0.2%) for each method. We observed that KIASORT detected ∼5% higher proportion of refractory units among those with high signal-to-noise ratio (SNR) than Kilosort4 (Fig. 5*E*). We need to emphasize that the Figure 5 is intended as a demonstration on real recordings and a qualitative sanity check, not as evidence of ground-truth superiority. Quantitative accuracy comparisons are based on simulated or benchmark datasets with known spike identities, whereas the results shown here serve only to illustrate KIASORT's performance on real data.

**Figure 6.**
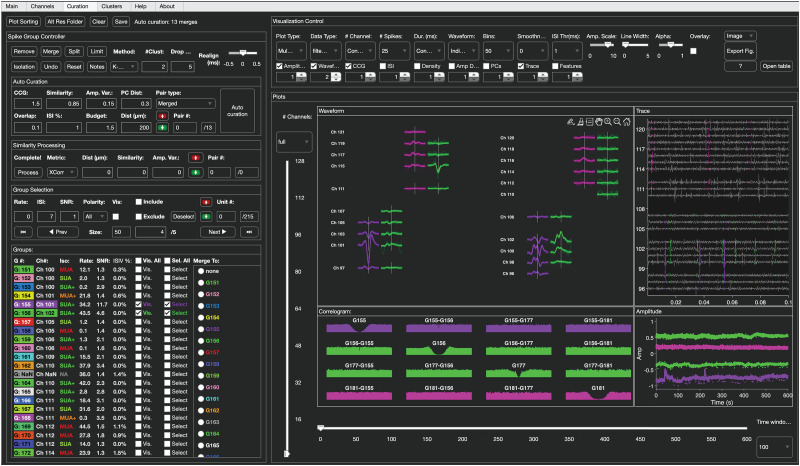
KIASORT GUI. KIASORT includes a GUI that provides a unified platform for data inspection, sorting, and post hoc curation. A screenshot of the curation tab which allows users to visualize sorted units; remove or merge units; change isolation types; perform pairwise waveform-similarity processing; and inspect clusters via cross-correlograms, ISI distributions, presence ratios, waveforms, and low-dimensional feature embeddings on each channel.

As another practical note, KIASORT includes a GUI that integrates both sorting and post hoc curation within a unified platform. This setup enables data exploration, user-defined parameter adjustments, and manual curation and verification of sorting results (Fig. 6). KIASORT is highly optimized to achieve speeds comparable to real-time processing benchmarks using only standard CPUs, making it accessible to users without high-end computational hardware while also supporting GPU acceleration for improved efficiency.

## Discussion

We introduced KIASORT, a novel spike-sorting algorithm for geometry-free tracking of neurons in high-channel recordings. KIASORT implements a per-neuron tracking system and uses a geometry-free approach to handle neuron-specific drift and nonlinear waveform changes that challenge existing spike-sorting algorithms. Our results demonstrate that KIASORT outperforms the current state-of-the-art method, Kilosort4, under conditions with per-neuron drift variability (Fig. 4*D*,*E*), which is common in real neurophysiological recordings (Fig. 5*D*).

One of the key innovations of KIASORT, compared with existing methods, is its hybrid use of nonlinear UMAP combined with linear PCA for dimensionality reduction (Fig. 2*A*). While previous approaches rely primarily on linear embeddings (Harris et al., 2000; Quiroga et al., 2004) with a priori assumptions about the geometry of the cluster feature space, integrating UMAP's nonlinear manifold preservation makes KIASORT better suited to arbitrarily shaped clusters and temporally changing waveforms. This hybrid approach can also handle complex drift manifolds that might otherwise be oversplit or merged inappropriately (Rossant et al., 2016; Hilgen et al., 2017). The advantage of the hybrid PCA + UMAP feature space becomes most pronounced when waveforms undergo nonlinear transformations likely arising from differential drift, voltage-dependent kinetics, or complex electrode-neuron geometry that produce curved manifolds in feature space. We showed that as the degree of nonlinearity increases, the UMAP component captures local manifold structure that PCA inherently misses, yielding progressively larger performance gains (Fig. S4*A*,*B*).

Another innovation and unique feature of KIASORT is its relaxation of probe-geometry assumptions in favor of a per-neuron, data-driven tracking approach that follows each neuron independently. Compared with earlier correction methods (Rossant et al., 2016), more recent approaches (from Kilosort2 onward) have implemented piecewise drift-tracking and correction models (Pachitariu et al., 2024); however, these methods still assume that neurons in close spatial proximity experience similar drift patterns and thus fail to account for drift arising from horizontal probe displacement, oblique insertion, or heterogeneous neural orientations. Our biophysical simulations demonstrate that even small deviations from one-dimensional motion can substantially distort waveforms in ways that might not be corrected by even perfect one-dimensional displacement models (Fig. 4*B*). KIASORT's hybrid clustering, channel-specific template/classifier construction, and neuron-based tracking system allow it to adapt to these complex patterns without imposing geometric constraints. The performance advantage is particularly pronounced when the variability of nonlinear waveforms or the heterogeneity of drift increases in the neural population (Fig. 4*D*,*E*), with KIASORT identifying 5–15% more high-quality units than Kilosort4. It is worth noting that in conditions that more closely match the geometric assumptions of template-based sorters, Kilosort4 shows a slight advantage, although KIASORT still outperforms the other methods tested under those same conditions (Fig. S4*E*).

The modular structure of KIASORT (Fig. 1) is another advantage, particularly for tracking neurons that remain consistently available across multiple recording sessions. KIASORT separates the sorting process into three distinct modules, allowing users to sample data and build channel-specific templates or classifiers on one dataset and then apply the learned model to additional data by running only the third module (sorting the data). This modular design is integrated into a user-friendly GUI with editing features that facilitate data inspection, spike sorting, and post hoc curation within a unified framework. This all-in-one platform is another development of KIASORT that is currently missing from existing spike-sorting pipelines (Buccino et al., 2020; Magland et al., 2020; Pachitariu et al., 2024).

Despite these advances, it is worth noting that, like any algorithm, KIASORT faces challenges and limitations that we outline here. A key challenge occurs when drift is so severe that a neuron completely vanishes and becomes indistinguishable from baseline noise, so that no spikes are detected on the reference channel. In such cases, the algorithm may split a single neuron into multiple units, which then might require post hoc manual curation and merging which can be done within the same sorting platform (Fig. 6). Another limitation is when neurons have very low firing rates (< 0.1 Hz), which might be missed entirely during the sampling phase and thus not identified in the clustering stage. Additionally, severe but transient noise conditions, such as electrical stimulation artifacts that saturate the amplifier and cannot be effectively removed, do not necessarily prevent spike detection but, if they occur frequently, can result in false-positive unit detections, which is addressable with post hoc manual curation.

In summary, KIASORT presents a novel and fundamentally restructured spike-sorting algorithm, showing significant advances in spike sorting for multichannel recordings, particularly for recordings with complex drift patterns. Its geometry-free design and per-neuron tracking approach make KIASORT less dependent on prior assumptions, making it suitable for nonlinear variabilities in waveform shapes and different probe design.

## Data availability

The whole pipeline and code for KIASORT is available from https://kiasort.com.
